# Large Shift in Symbiont Assemblage in the Invasive Red Turpentine Beetle

**DOI:** 10.1371/journal.pone.0078126

**Published:** 2013-10-18

**Authors:** Stephen J. Taerum, Tuan A. Duong, Z. Wilhelm de Beer, Nancy Gillette, Jiang-Hua Sun, Donald R. Owen, Michael J. Wingfield

**Affiliations:** 1 Department of Genetics, Forestry and Agricultural Biotechnology Institute, University of Pretoria, Pretoria, South Africa; 2 Department of Microbiology and Plant Pathology, Forestry and Agricultural Biotechnology Institute, University of Pretoria, Pretoria, South Africa; 3 Pacific Southwest Research Station, United States Department of Agriculture Forest Service, Albany, California, United States of America; 4 State Key Laboratory of Integrated Management of Pest Insects and Rodents, Institute of Zoology, Chinese Academy of Sciences, Beijing, China; 5 California Department of Forestry and Fire Protection, Redding, California, United States of America; Emory University, United States of America

## Abstract

Changes in symbiont assemblages can affect the success and impact of invasive species, and may provide knowledge regarding the invasion histories of their vectors. Bark beetle symbioses are ideal systems to study changes in symbiont assemblages resulting from invasions. The red turpentine beetle (*Dendroctonus valens*) is a bark beetle species that recently invaded China from its native range in North America. It is associated with ophiostomatalean fungi in both locations, although the fungi have previously been well-surveyed only in China. We surveyed the ophiostomatalean fungi associated with *D. valens* in eastern and western North America, and identified the fungal species using multi-gene phylogenies. From the 307 collected isolates (147 in eastern North America and 160 in western North America), we identified 20 species: 11 in eastern North America and 13 in western North America. Four species were shared between eastern North America and western North America, one species (*Ophiostoma floccosum*) was shared between western North America and China, and three species (*Grosmannia koreana*, *Leptographium procerum*, and *Ophiostoma abietinum*) were shared between eastern North America and China. *Ophiostoma floccosum* and *O. abietinum* have worldwide distributions, and were rarely isolated from *D. valens*. However, *G. koreana* and *L. procerum* are primarily limited to Asia and North America respectively. *Leptographium procerum*, which is thought to be native to North America, represented >45% of the symbionts of *D. valens* in eastern North America and China, suggesting *D. valens* may have been introduced to China from eastern North America. These results are surprising, as previous population genetics studies on *D. valens* based on the cytochrome oxidase I gene have suggested that the insect was introduced into China from western North America.

## Introduction

Symbionts greatly influence the success and impact of many human-mediated species invasions [1-4]. Mutualistic symbionts can facilitate invasive species or enhance the damage they cause (i.e., invasional meltdown [5]). For example, successful plant invasions often depend on co-invading or newly adopted mychorrhizae [6] or endophytes [7-9], while plants that lose their mutualists can have lowered fitness in invaded environments [10]. Invasive forest insects can also benefit from microbial symbionts that allow the insects to aggressively colonize naive hosts [11]. Alternatively, invasive species can benefit from the loss of co-evolved parasites or pathogens (i.e., enemy release [12,13]), while parasites and pathogens that are present in the invaded ranges can inhibit invasive species [14]. Finally, some coinvading commensalists, mutualists, and pathogens can indirectly influence the success of invasive species by outcompeting native symbionts that would reduce the invader’s fitness, or by inhibiting the invader’s competitors or predators [15,16].

Changes in individual symbiont species during invasions have received increased attention in recent years, especially in the cases of invasive plants [1,17], insects [11], and marine organisms [18]. However, studies of changes in entire symbiont assemblages are more rare [19-21]. Comparisons between symbiont assemblages in a vector’s native and invasive ranges may be useful for clarifying the origin and invasion history of their vectors, especially where molecular data provide unclear results. As symbiont communities can vary substantially over a species' range [22-24], more symbionts should be shared between the invasive population and its source population, assuming the symbionts were vectored and successfully established in the invaded environment. Changes in symbiont assemblage over time in the invaded environment may also reflect the time elapsed since invasion and how an invader spread, as the invader should obtain a greater number and a wider variety of native symbionts over time and as it spreads. These changes may be informative regarding the traits that make certain symbionts successful invaders, and the traits that allow other symbionts to jump onto invasive species.

Invasive insect symbioses in forest ecosystems are ideal for the study of changes in symbiont assemblages. Several insect species have invaded naive forest ecosystems, sometimes causing significant ecological and economic damage [25,26]. Forest insects are often associated with a variety of microbial and animal symbionts, which can coinvade environments with their vectors, causing significant damage to the invaded forests [11,27]. In addition, symbiont assemblages of forest insects can change in invaded environments [20], potentially increasing the damage caused by invasive forest insect symbioses.

Bark beetles (Coleoptera: Curculionidae: Scolytinae) and their fungal symbionts represent a large percentage of invasive insect symbioses [11]. These insects feed and reproduce in the inner bark of trees [28], while vectoring a diverse assemblage of fungi between their hosts [29,30]. Although the vast majority of bark beetle species colonize only dead or dying trees in their native environments, some can aggressively attack and kill healthy trees [31]. Some invasive bark beetle species switch from non-aggressive to aggressive tree-killing life histories in their invaded environments, posing an additional challenge for researchers seeking to alleviate the effects of invasive species on forest ecosystems [11]. In addition to being major pests, several bark beetle species provide model systems to study host-symbiont interactions [32], making them ideal to investigate the effects of invasions on symbiont assemblages.

Among the most common and important fungal associates of bark beetles are a monophyletic group of fungi in the order Ophiostomatales (Ascomycota) [33-35], here-in referred to as ophiostomatalean fungi. These fungi have evolved traits that facilitate their transmission between host trees by bark beetle vectors [34,36]. Many of these fungi are externally acquired from the trees in which the bark beetles develop. The fungal spores attach to the exoskeletons of adult bark beetles or mites that are in turn vectored by the beetles [36]. Some of the fungi are transported in specialized structures on the bark beetles that are referred to as mycangia [36]. Although the majority of ophiostomatalean fungi are benign or mildly pathogenic to their host trees [37], some bark beetle-vectored ophiostomatalean fungi are tree-killing pathogens [34]. Ophiostomatalean symbionts also vary in their association with their bark beetle vectors [38]. Most ophiostomatalean fungi are casual and occasional commensalists [39], while a few provide nutrition for the larvae of bark beetle vectors [40,41]. Still others can inhibit bark beetle brood development, leading to negative feedback effects on the vector populations [42]. Some ophiostomatalean fungi have also been hypothesized to assist their bark beetle vectors in overcoming host tree defenses [43,44], although this hypothesis has been recently challenged [37]. As most ophiostomatalean symbionts of bark beetles are externally transported and casual associates, invasive bark beetle species are likely to lose some of their previous fungal symbionts, while acquiring new symbionts in the invaded environment.

Positive and negative interactions between bark beetles and symbiotic ophiostomatoid fungi can mediate the ability of bark beetles to form tree-killing epidemics [30,45]. For invasive bark beetles, of which there are many examples [46], symbiotic fungi may exacerbate the effects of the insect vectors even if the fungi are non-aggressive or commensalists in their native environments. For example, there is evidence that the red turpentine beetle (*Dendroctonus valens* LeConte) recently became an aggressive tree-killer in its invaded range in part because of interactions with its ophiostomatalean fungus symbionts [47,48].

**Figure 1:**
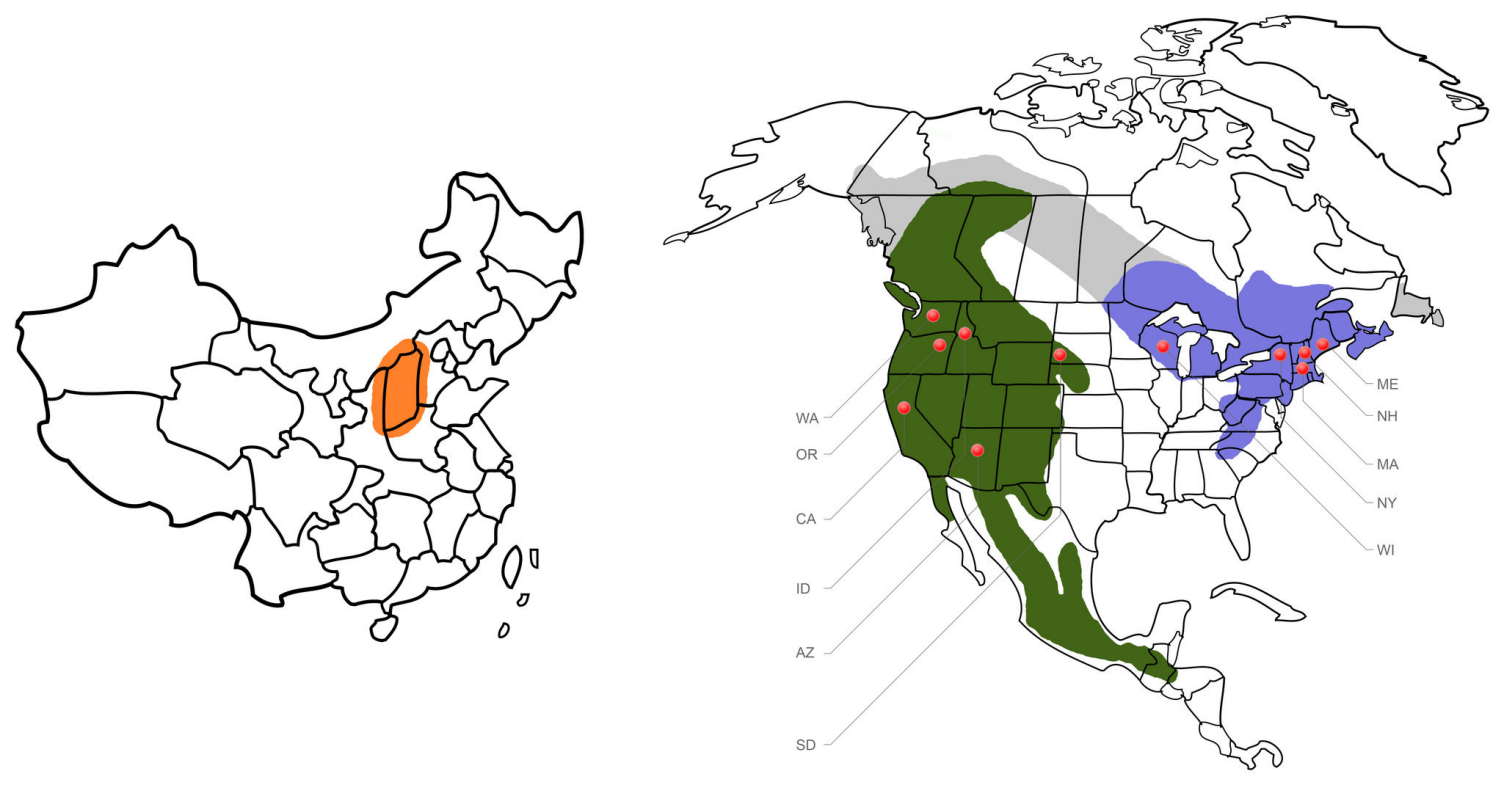
Ranges of *Dendroctonus valens* and study collection locations. Ranges of *D. valens* in China (orange), western North America (green) and eastern North America (blue), based on Yan et al. [50] and Wood et al. [28]. The potential range of *D. valens* is shaded grey (based on Owen et al. [49]). Collection locations in North America are indicated by red dots. State names are as follows: AZ = Arizona, CA = California, ID = Idaho, ME = Maine, MA = Massachusetts, NH = New Hampshire, NY = New York, OR = Oregon, SD = South Dakota, WA = Washington, and WI = Wisconsin.

*Dendroctonus valens* is native to North America, where its range extends from southeastern Alaska to Honduras (Figure 1 [28,49]). The range is effectively divided into two populations separated by the Great Plains and the spruce- and poplar-dominated boreal forest: eastern and western North America (ENA and WNA respectively). However, some researchers have suggested that the range may in fact be continuous through the boreal forest [49]. Although there may be some movement of *D. valens* between ENA and WNA, for this study we will consider the two populations to be separate, as there is most likely little dispersal between ENA and WNA because of the few pine stands in the North American boreal forest, and climate conditions that are highly unfavorable to the survival of *D. valens* there.

*Dendroctonus valens* is considered a minor pest in North America, as it typically colonizes dying or stressed pine trees [49]. In the 1980’s, *D. valens* invaded China (Figure 1), where it aggressively kills pine species native to China [50]. Population genetics studies on *D. valens* using cytochrome oxidase I (COI) led to the suggestion that *D. valens* was most likely introduced from the Pacific Northwest in WNA [51,52]. However, both studies included only one population of *D. valens* from ENA, in the U.S. state of Michigan. Because *D. valens* is widespread in ENA, one population could have represented an insufficient sampling to draw conclusions regarding the origin of *D. valens*. In addition, the *D. valens* genome contains several COI pseudogenes that may have decreased the accuracy of the previous population genetic studies [53].

*Dendroctonus valens* is associated with ophiostomatalean fungi in both North America [32] and China [54,55]. The ophiostomatalean symbionts of *D. valens* have been well-surveyed in China, with 193 strains isolated and identified from several locations within *D. valens*’ Chinese range [54,55]. However, far less is known about the ophiostomatalean symbionts of *D. valens* in North America, and which (if any) may have coinvaded China with *D. valens*. A greater number of ophiostomatalean species should be shared between China and the *D. valens* population of origin in North America. In addition, the changes in ophiostomatalean community are important because one ophiostomatalean species, *Leptographium procerum*, is hypothesized to have contributed to the aggressive behavior of *D. valens* in China [48]. *Leptographium procerum*, which is non-pathogenic in its native range in North America [56,57] (however, see Alexander et al. [58]), may be pathogenic in China as it can kill seedlings of *Pinus tabuliformis*, the primary host of *D. valens* in China [47,48]. *Leptographium procerum* most likely coinvaded China along with *D. valens*, as it is a known associate of *D. valens* in North America [59], and has not been found in China except in association with *D. valens* [60]. *Pinus tabuliformis* produces higher amounts of the monoterpene, 3-carene, when infected by pathogenic strains of *L. procerum* [47,48]. As 3-carene is the strongest attractant of *D. valens* [61,62], the increased 3-carene production may represent a feedback mechanism that increases the aggressive behavior of *D. valens* in China. Although this hypothesis is not definitive, the pathogenicity of *L. procerum* in China most certainly contributes to the damage caused by *D. valens*.

In this study, we used phylogenetic methods to compare the ophiostomatalean symbiont assemblages of *D. valens* in ENA and WNA with those in China. We hypothesized that more symbiont species should be shared between WNA and China, based on the predicted WNA origin of *D. valens* introduced to China. The overall aim was thus to demonstrate whether changes in symbiont assembly in invasive species reflect the findings of molecular studies of the vectors, and possibly predict which symbionts are more likely to be successfully vectored during future invasion events.

## Methods

### Ethics statement

This study did not involve organisms protected by federal, state or local law. Collections made by Forest Service employees on National Forests are categorically excluded from permit requirements as long as they are limited in extent, are for research purposes, and involve organisms that are not protected by federal, state, or local law [63]. Collectors were given verbal permission to collect in: 1) Ware, Massachusetts, by the Department of Conservation and Recreation - Massachusetts; 2) Fort Drum, New York, by the Forest Management Group at Fort Drum; 3) the Colville Reservation, Washington, by the Confederated Tribes of the Colville Reservation; and 4) Wood County, Wisconsin, by the Plum Creek Timber Company.

### Collections and isolations

Between 2008 and 2011, we collected *D. valens* adults and parts of their galleries from several locations in WNA (Arizona, California, Idaho, Oregon, South Dakota, and Washington; Figure 1; for specific collection and storage details for each location see Table S1) and ENA (Maine, Massachusetts, New Hampshire, New York and Wisconsin). Bark beetle adults were collected either by hand from trees colonized by *D. valens* or with funnel traps near infested trees. Bark beetles were stored at -20°C or -80°C and galleries at 4°C until fungal isolations, which were made within two weeks after collection. Bark beetles were rolled onto 2% malt extract agar (MEA; 20 g agar and 20 g malt extract per 1 L water) containing 0.5 g cycloheximide, which is selective for fungi in the order Ophiostomatales. Gallery isolations were also conducted using the aforementioned selective media. Fungi were incubated at 20°C, then subcultured onto 2% MEA and stored at 4°C. Only one representative of each fungal species was counted per beetle or gallery to ensure that replicates of each fungal species were independent.

### DNA extraction, PCR, and sequencing

Fungal cultures were sorted into two major groups based on morphology: *Ophiostoma sensu lato* (which we analyzed with the ophiostomatalean genera *Fragosphaeria*, *Ceratocystiopsis*, and *Graphilibum*) and *Leptographium sensu lato* [64]. DNA extractions from representative samples of each group were performed following the methods of Duong et al. [65]. For *Leptographium sensu lato*, we amplified a part of the β-tubulin gene (βt), the elongation factor-1 alpha gene (EF), and the internal transcribed spacer 2 region and a part of the large subunit (ITS2-LSU) of the ribosomal DNA. For *Ophiostoma sensu lato*, we amplified a part of βt and the internal transcribed spacers 1 and 2 (ITS1-ITS2) of the ribosomal DNA. βt was amplified using primers Bt2a and Bt2b [66], EF was amplified using primers EF1F and EF2R [67], ITS2-LSU was amplified using primers ITS3 and LR3 [68], and ITS1-ITS2 was amplified using primers ITS1-F [69] and ITS4 [68]. The thermal cycler protocol and sequencing were as described by Duong et al. [65].

We compared the ITS1-ITS2 sequences with those of 87 closely related species downloaded from Genbank: 76 strains representing *Ophiostoma sensu lato* (including six from China; [54,55]), and 11 strains of *Leptographium sensu lato* as an outgroup. We compared the ITS2-LSU sequences with those of 69 closely related species downloaded from Genbank: 65 strains from *Leptographium sensu lato* (including seven from China), and four strains of *Ophiostoma sensu lato* as an outgroup. The study organisms were placed in previously described species complexes [64], based on the ITS1-ITS2 and ITS2-LSU phylogenies. Where a sample did not belong to a species complex, it was analyzed along with its closest species complex. We sorted the βt and EF sequences of species belonging to *Leptographium sensu lato* into the *Grosmannia aurea*, *G. galeiformis* and *G. olivacea*, *L. lundbergii* and *G. huntii*, and *L. procerum* species complexes, and we sorted the βt sequences of species belonging to *Ophiostoma sensu lato* into the *Ophiostoma ips*, *O. piceae* and *O. minus*, and *Sporothrix schenckii*-*O. stenoceras* species complexes. The sequences obtained in this study were submitted to GenBank (accession numbers KF515849-KF515917). The following numbers of sequences were downloaded from Genbank for comparison with the study sequences: for *G. aurea*, 15 for βt and 16 for EF; for *G. galeiformis* and *G. olivacea*, 14 for βt and 11 for EF; for *L. lundbergii* and *G. huntii*, 22 for βt (including four from China) and 20 for EF (including two from China); for *L. procerum*, 24 for βt (including nine from China) and 25 for EF (including 11 from China); for *O. ips*, 11 for βt; for *O. piceae* and *O. minus*, 24 for βt (including three from China); and for *S. schenckii*-*O. stenoceras*, 14 for βt (including one from China).

### Phylogenetic analyses

We aligned the sequences using MAFFT 6 (http://mafft.cbrc.jp/alignment/software/ [70]). For maximum likelihood (ML) analyses, we determined the substitution models using jModelTest 0.1.1 [71] (Table S2). We then conducted maximum likelihood analyses using PhyML 3.0 for the PC [72], and obtained bootstrap support using 1000 maximum likelihood replicates.

We conducted Bayesian analyses using MrBayes 3.1.2 [73] and a Markov chain Monte Carlo analysis. Evolutionary models were determined for each dataset using jModelTest 0.1.1 [71]. Four MCMC chains were run with 5000000 generations each. The program Tracer 1.4 [74] was used to determine the burn-in values, and we discarded the trees sampled in the burn-in phase. One tree out of every 100 generations was sampled to calculate the posterior probabilities (PP) at each node. The PP values were added to the ML trees.

## Results

### Isolations and culture deposition

We isolated 307 ophiostomatalean isolates in total: 160 from WNA (six from Arizona, 143 from California, one from Idaho, two from Oregon, three from South Dakota, and five from Washington) and 147 from ENA (37 from Maine, 43 from Massachusetts, 31 from New Hampshire, two from New York, and 34 from Wisconsin). Representative isolates were deposited in the culture collection of the Forestry and Agricultural Biotechnology Institute (FABI) in Pretoria, South Africa, and given CMW numbers (i.e., isolate identifiers). Ninety-three strains from WNA were of *Leptographium sensu lato* and 67 resided in *Ophiostoma sensu lato*. In contrast, 130 strains from ENA were of *Leptographium sensu lato* and 17 were of *Ophiostoma sensu lato*.

### Sequences and phylogenetic results

The substitution models and burn-in values are summarized in Table S2. The ITS1-ITS2 dataset had 841 aligned base pairs (BP), of which 550 were variable, and the ITS2-LSU dataset had 650 aligned BP (220 variable). For the βt datasets, there were 373 aligned BP (15 variable) in the *G. aurea* complex, 284 aligned BP (94 variable) in the *G. galeiformis* and *G. olivacea* complexes, 384 aligned BP (63 variable) in the *L. lundbergii* and *G. huntii* complexes, 375 aligned BP (59 variable) in the *L. procerum* complex, 481 BP (203 variable) in the *O. ips* complex, 684 BP (460 variable) in the *O. piceae* and *O. minus* complexes, and 321 BP (90 variable) in the *S. schenckii*-*O. stenoceras* complex. For the EF gene regions, there were 518 BP (20 variable) in the *G. aurea* complex, 669 BP (265 variable) in the *G. galeiformis* and *G. olivacea* complexes, 690 BP (102 variable) in the *L. lundbergii* and *G. huntii* complexes, and 727 BP (175 variable) in the *L. procerum* complex.

**Figure 2:**
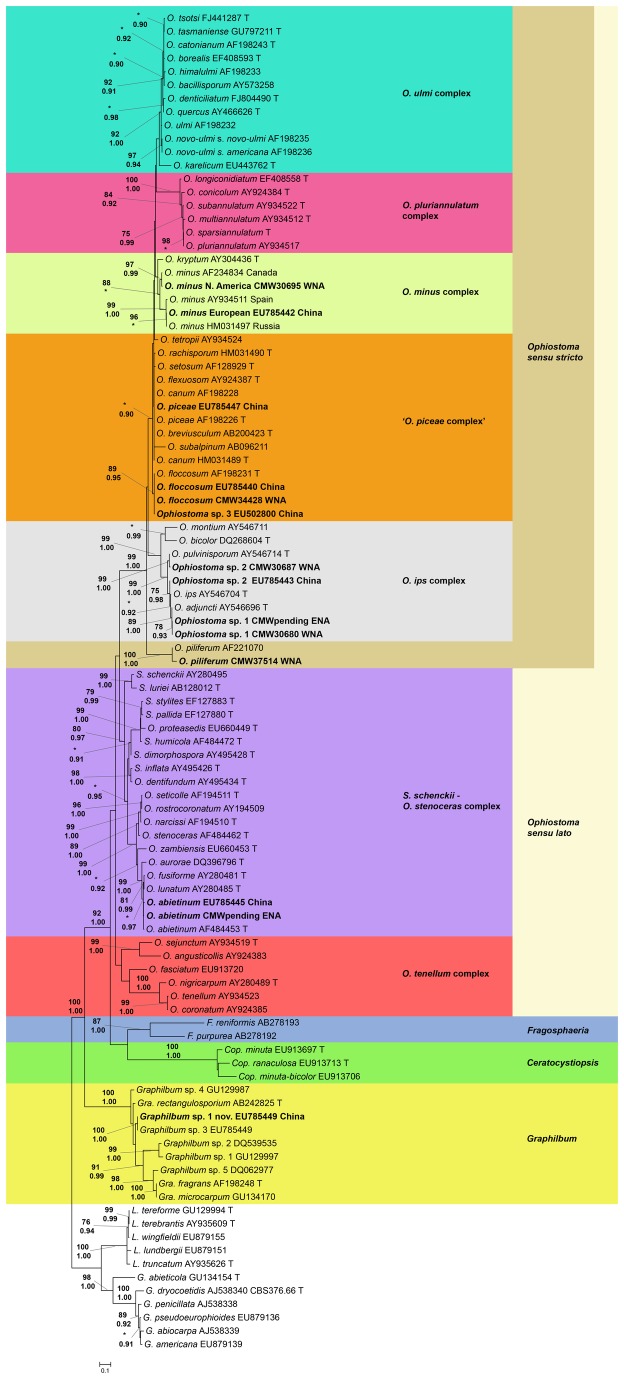
Phylogram of *Ophiostoma sensu* *lato* based on ITS1-ITS2. Maximum likelihood (ML) phylogram of 75 ophiostomatalean fungi in the genus *Ophiostoma sensu* *lato*, two in the genus *Fragosphaeria*, three in the genus *Ceratocystiopsis*, nine in the genus *Graphilbum*, and 11 in the genus *Leptographium sensu* *lato* as an outgroup, based on ITS1-ITS2. Each strain is indicated by its species name, the Genbank accession number or CMW culture collection number (if accession number is not available), and a T if the isolate originates from a species’ type specimen. Strains associated with *D. valens* either from this study or the Chinese studies [54,55] are in bold font, and are followed with the location they were isolated from. Strains are subdivided into species complexes indicated by different colors. Statistical support is given to the left of the nodes, with ML bootstrap proportions on top (only values greater than 75 are shown), and Bayesian posterior probability (PP) values on the bottom (only values greater than 0.90 are shown). * indicates that the ML or PP values were not significant at those nodes.

**Figure 3:**
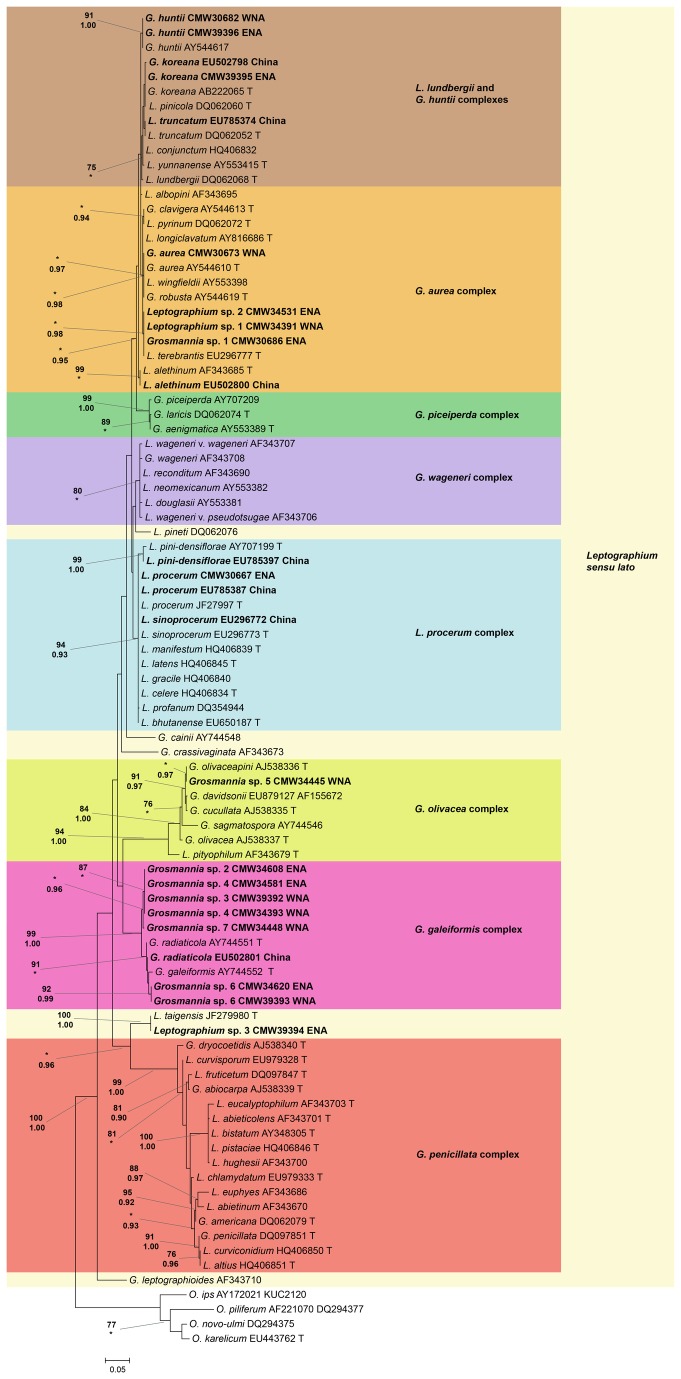
Phylogram of *Leptographium sensu* *lato* based on ITS2-LSU. ML phylogram of 87 ophiostomatalean fungi in the genus *Leptographium sensu* *lato*, and four in the genus *Ophiostoma sensu* *lato* as an outgroup, based on ITS2-LSU. Each strain is indicated following the same criteria as Figure 2. Statistical support for the nodes is shown in the same format as Figure 2.

Based on phylogenetic analyses, a total of 20 ophiostomatalean species symbiotic with *D. valens* in North America were found (30 species including the symbionts of *D. valens* in China; Figures 2,3). The phylogenies generated using βt and EF sequences supported the taxonomic placement of the ITS1-ITS2 and ITS2-LSU phylogenies (Figures S1-S7). There were 13 species associated with *D. valens* in WNA and 11 species associated with *D. valens* in ENA, compared with 15 species associated with *D. valens* in China (Table 1). No species were shared between all three locations. Eight species from WNA and seven species from ENA represented undescribed taxa. All of the isolated species belonged to well-supported species complexes, except for *Leptographium* sp. 3, which is closely related to *L. taigensis* and was analyzed along with the species in the *G. galeiformis* and *G. olivacea* species complexes, and *O. piliferum*, which was analyzed along with the species in the *O. piceae* and *O. minus* complexes. The ophiostomatalean assemblages from ENA and WNA were very distinct. Here, only four species (*G. huntii*, *Grosmannia* sp. 4, *Grosmannia* sp. 6, and *Ophiostoma* sp. 1) were shared between ENA and WNA. Few species were shared between China and North America and these included one species (*O. floccosum*) shared between WNA and China, and three species (*G. koreana*, *L. procerum*, and *O. abietinum*) shared between ENA and China.

**Table 1:** Numbers of isolates of each Ophiostomatalean species collected in ENA, WNA, and China.

Species	Total ENA^1^	Total WNA	Total China [54,55]
*Graphilbum* sp.	0	0	3 (1.5)
*Grosmannia* sp. 1	5 (3.4)	0	0
*Grosmannia* sp. 2	8 (5.4)	0	0
*Grosmannia* sp. 3	0	1 (0.6)	0
*Grosmannia* sp. 4	10 (6.8)	10 (6.3)	0
*Grosmannia* sp. 5	0	2 (1.3)	0
*Grosmannia* sp. 6	2 (1.3)	4 (2.5)	0
*Grosmannia* sp. 7	0	3 (1.9)	0
*G. aurea*	0	13 (8.1)	0
*G. huntii*	8 (5.4)	2 (1.3)	0
*G. koreana*	6 (4.1)	0	11 (5.4)
*G. radiaticola*	0	0	5 (2.4)
*Leptographium* sp. 1	0	58 (36.3)	0
*Leptographium* sp. 2	23 (15.6)	0	0
*Leptographium* sp. 3	1 (0.7)	0	0
*L. alethinum*	0	0	6 (2.9)
*L. pini-densiflorae*	0	0	3 (1.5)
*L. procerum*	67 (45.6)	0	125 (61.0)
*L. sinoprocerum*	0	0	12 (5.9)
*L. truncatum*	0	0	9 (4.4)
*Ophiostoma* sp. 1	16 (10.9)	53 (33.1)	0
*Ophiostoma* sp. 2	0	1 (0.6)	0
*Ophiostoma* sp. 3	0	0	2 (1.0)
*O. abietinum*	1 (0.7)	0	3 (1.5)
*O. floccosum*	0	8 (5.0)	16 (7.8)
*O. ips*	0	0	7 (3.4)
*O. minus* (Europe)	0	0	1 (0.5)
*O. minus* (N. America)	0	1 (0.6)	0
*O. piceae*	0	0	2 (1.0)
*O. piliferum*	0	4 (2.5)	0
Total # isolates per location	147	160	205
Total # species	11	13	15

1 The numbers of isolates are followed by the percentage of total isolates that species represents in each location.

The frequency of the different fungal species found from each of the states sampled is summarized in Table S3. Between one and seven species were isolated in each state of ENA. Several species were found in multiple states, especially *L. procerum* and *Ophiostoma* sp. 1, which were found in four states (Maine, Massachusetts, New Hampshire, and Wisconsin), and *Leptographium* sp. 2, which was found in three states (Maine, Massachusetts, and New Hampshire). *Leptographium procerum* represented between 25.8% and 88.2% of collected strains in the states from which they were collected, while *Ophiostoma* sp. 1 represented between 2.9% and 23.3%, and *Leptographium* sp. 2 represented between 6.5% and 40.5%. *Grosmannia* sp. 2, *Grosmannia* sp. 6, *G. huntii*, *Leptographium* sp. 3, and *O. abietinum* were each found in only one of the five ENA states, although *Grosmannia* sp. 2 and *G. huntii* represented large percentages of the species isolated in the states from which they were found (25.8% for *Grosmannia* sp. 2 in New Hampshire, and 18.6% for *G. huntii* in Massachusetts).

Between one and 10 species were isolated from each state in WNA. *Grosmannia aurea*, and *Ophiostoma* sp. 1 were found in five of the six states (*G. aurea* was not isolated in Idaho, and *Ophiostoma* sp. 1 was not found in Oregon). Those species represented between 3.5% and 66.7%, and between 16.7% and 100% respectively of their states’ isolates. *Grosmannia huntii* was the only other species isolated that occurred in multiple states in WNA (Oregon and Washington), representing 20% to 50% of the isolates from those states. Most of the species that were isolated in only one state were found in California, with only one isolate of *Ophiostoma* sp. 2 in Arizona and one isolate of *O. minus* (North America) in South Dakota the only exceptions. All of the species found only in California represented <10% of the state’s isolates, except for *Leptographium* sp. 1, which represented 40.6% of the isolates from that state.

## Discussion

Based on our analyses, there were strong differences in ophiostomatalean symbiont assemblages of *D. valens* between ENA, WNA and China. These differences were supported by vigorous phylogenetic and statistical analyses on multiple gene regions. In addition, several new ophiostomatalean species were discovered in this study. Although few ophiostomatalean species were shared between the three populations, more species were shared between ENA and China than between WNA and China.

Collectively, this study and those of Lu et al. [54,55] have resulted in a collection of 500 isolates of ophiostomatalean fungi from *D. valens*. Of these, 193 are from the invasive range of the beetle in China, 160 are from WNA, and 147 are from ENA. These represent 30 different species of ophiostomatalean fungi. While this represents a large collection and a substantial biodiversity for a single bark beetle species, it is clear that the numbers of isolates of the various fungi most likely represent only a partial representation of their relative abundance. This is due to the fact that isolation success from beetles on agar is dependent on many variable conditions including competition between the ophiostomatalean fungi and contaminant microbes including bacteria and other fungi. Furthermore, the occurrence of symbionts on the beetles is not uniform and is dependent on those that sporulate most effectively in the particular galleries from which they are collected. In addition, collection and isolation methods varied between locations and by collectors, potentially influencing the observed species frequencies in the study locations. Nevertheless, sampling in this study was intensive in both WNA and ENA and we believe that the results of this study at least provide a relatively comprehensive view of the fungi associated with *D. valens* in the areas considered.

Only four species of ophiostomatalean fungi were shared between ENA and WNA, suggesting that the movement and establishment of symbionts between the two *D. valens* populations is rare. Similarly, Adams et al. [22] found that the communities of actinomycete bacteria symbiotic with *D. valens* in ENA were very distinct from those associated with *D. valens* in WNA, although they sampled from only one population in ENA (in the state of Wisconsin). Our findings may support the effective separation of *D. valens*’ range into ENA and WNA, as symbionts would be more likely to be shared if there was continuous movement amongst the locations (akin to gene flow). Alternatively, differences in the abiotic environment, host tree species, or the phenotype of *D. valens* between ENA and WNA might explain the different ophiostomatalean assemblages in these two areas. Further sampling in Canada, especially in British Columbia and the pine stands in the boreal forest, should demonstrate whether there is a distinct cut-off in symbiont assemblages between the ENA and WNA populations, whether there is a transition zone including more shared symbionts from ENA and WNA, or whether *D. valens* in Canada has its own assemblage adapted to boreal North America.

There was a great deal of diversity between states within ENA and WNA, although several species were shared between the different states. States in ENA were typically dominated by *L. procerum*, which occurred in four of the five states, and represented at least 25% of the isolates from each of those states. *Ophiostoma* sp. 1 and *Leptographium* sp. 2 were also widespread in ENA, although they were much less frequently encountered than *L. procerum*. States in WNA were dominated by *Ophiostoma* sp. 1, which represented at least 15% of the isolates collected in the five states where it was found. *Grosmannia aurea* was also widespread, although isolated much less frequently. Much of the difference among states may be because of variation in the number of isolates collected at each location, as more species tended to be isolated with increasing numbers of collected isolates. This was especially true in WNA, where 89% of the isolates were collected in California, yielding 10 of the 13 species found in WNA. However, most of the species isolated in California were rare, suggesting that they are only occasional or casual associates of *D. valens*.

A few species that were isolated in only one or two states made up a large percentage of the isolates in those states. For example, *Grosmannia* sp. 2, *Grosmannia* sp. 4, and *G. koreana* represented 25.8%, 16.1% and 16.1% respectively of the isolates in New Hampshire, *Grosmannia* sp. 4 and *G. huntii* represented 11.6% and 18.6% respectively of the isolates in Massachusetts, and *Leptographium* sp. 1 represented 40.6% of the isolates in California. In addition, *G. huntii* represented a large percentage of isolates from Oregon and Washington (50% and 20% respectively), and *Ophiostoma minus* (North America) represented a large percentage of isolates in South Dakota (33.3%), but this may represent an artifact of the low numbers of isolates from those states. Yet some variation in symbiont assemblage is expected, as there is a great deal of environmental variation in *D. valens*’ range in ENA and WNA, so there may also be geographical variation in *D. valens*’ phenotype [75]. For example, Adams et al. [22] discovered variation in actinomycete symbionts associated with *D. valens*, even between geographically close sites, suggesting that different environments favor different species assemblages. In addition, some of the variation may be caused by annual or seasonal variation in symbiont assemblage, as collections were made over four years. Additional sampling over time and in additional locations would provide more resolution to the spatial and temporal variation in symbiont assemblages.

Only one ophiostomatalean species from western North America and three species from eastern North America were shared with China, demonstrating that most ophiostomatalean associates did not coinvade with *D. valens*. This could be because there were insufficient propagules for establishment in China, or they could have been outcompeted by native ophiostomatalean fungi in China. All of the ophiostomatalean fungi associated with *D. valens* in China have been reported from Asia or Eastern Europe [60,76-84], suggesting that many of the symbionts were acquired through “vector-jumps,” whereby *D. valens* began vectoring ophiostomatalean fungi already present in host trees. These newly acquired symbionts may have been vectored by other beetle species or mites that colonized the trees along with *D. valens*.

The species shared between China and WNA (*O. floccosum*) is associated with a large variety of bark beetle species found worldwide [85], and it represented a small percentage of the fungi isolated from *D. valens* in China and WNA (0 to 12.0% and 5.0% respectively). Similarly, *O. abietinum*, which is shared between China and ENA, is cosmopolitan, symbiotic with a wide diversity of bark beetles [82,85-87], and is an occasional associate of *D. valens* in China and ENA (0 to 2.3% and 0.7% respectively). In contrast, the other two species shared between China and ENA, *G. koreana* and *L. procerum*, have much more limited ranges. *Grosmannia koreana* is primarily an Asian species [76,88], with this study being the first report of *G. koreana* from North America. The species represented 1.5 to 15% of the associates in China, and 4.1% of the symbionts in ENA. As *G. koreana* is prevalent in Asia, it may be more likely that *G. koreana* was adopted by *D. valens* in China. Conversely, *L. procerum* is primarily a North American species, although it has been introduced to China (most likely with *D. valens*), Europe, New Zealand, and South Africa [54,55,57]. There have been recent reports of *L. procerum* associated with the bark beetles *Tomicus piniperda* and *Ips sexdentatus* in Poland [81,82], although the fungus was isolated from only 3.3% and 2%, respectively, of the beetles collected, and was identified based on morphology only [81] or DNA sequences for only a single gene region [82].

*Leptographium procerum* was the most frequently isolated species in China [54,55] and ENA (51.7 to 70.7% and 45.6% respectively). The high percentage of *L. procerum* strains isolated from ENA in this study suggests that if *D. valens* invaded China from ENA it would have vectored a large number of propagules of this fungus. This would have resulted in a higher probability of successful establishment of *L. procerum* in China than any of the other ophiostomatalean symbionts of *D. valens*. All evidence emerging from this study suggests that *L. procerum* was introduced into China together with *D. valens*, especially considering that *L. procerum* has only been found in China as an associate of *D. valens*. Furthermore, the evidence strongly supports the notion that the source would not have been from WNA where large collections of the beetle have failed to yield the fungus, and rather from an area in ENA where *L. procerum* is commonly associated with the beetle. This study is in contrast to the view from previous studies based on COI [51,52] that *D. valens* was most likely introduced into China from WNA. Clearly, further population genetics studies including larger numbers of samples of *D. valens* from ENA will be needed to clarify the invasion history of *D. valens*.

The observed differences in symbiont assemblage revealed in this study may be at least in part due to characteristics of the symbionts involved. All of the ophiostomatalean fungi associated with *D. valens* are external symbionts, and are not transmitted vertically to offspring. Internal symbionts, such as gut microbes in animals [19,89], fungi and bacteria in specialized structures such as mycangia [32,90], and bacteria inhabiting root nodules [21], may have a better chance of retention during an invasion event because they are contained within their vectors. In addition, all of the associates of *D. valens* analyzed in this study are horizontally transmitted. Vertically transmitted symbionts are more likely to be vectored to new environments than horizontally transmitted symbionts [15,91]. Other characteristics, such as differences in the ability of symbionts to survive and reproduce in new environments with new competitors, may have influenced the ability of the ophiostomatalean symbionts to travel to and colonize China. Comparisons of different invasive symbioses where the symbionts have different characteristics (e.g., internal vs. external symbionts, vertically- vs. horizontally-transmitted symbionts, mutualists vs. parasites vs. commensalists, etc.) should confirm the importance of these characteristics to observed changes in symbiont assemblages.

The results of this study suggest that changes in symbiont assemblage may provide useful models to address many intriguing questions relating to invasion biology. Although molecular techniques are generally effective for addressing questions such as the origin of invasive species, the number of invasion events, the diversity of invasive populations, and the ways invasive populations spread and evolve [92-94], symbiont assemblage may prove useful where molecular data are inadequate, give ambiguous results, or are unavailable due to time or monetary constraints. Further work on changes in symbiosis assemblages with other invasive species should determine to what extent symbiont community membership is a useful trait to study in invasion biology. Intrinsic to such comparisons of symbiont assemblages is the requirement for baseline data on existing symbiotic associations in the native regions of the vectors. Such information is sorely lacking for many regions, as exemplified in the case of *D. valens* in North America. The information developed in this study has vastly increased our knowledge of the existing symbioses between *D. valens* and ophiostomatalean fungi, enhancing the possibility that we can detect new associations in the future. Expanded surveys of this type will be crucial for future biosecurity efforts [27].

## Supporting Information

Figure S1 **Phylogram of the *Ophiostomaips* species complex based on βt.** ML phylogram of 15 fungal isolates in the O. *ips* species complex, based on βt. Individual strains are indicated by their species name, followed by their Genbank accession number or CMW culture collection number (if accession number is not available), and a T if the isolate originates from a species’ type specimen. Isolates associated with *D. valens* that were collected in this study or in the Chinese studies [54,55] are followed by the locations they were isolated from in different colors: blue for ENA, green for WNA, and orange for China. Statistical support is given to the left of the nodes, with ML bootstrap proportions on top (only values greater than 75 are shown), and Bayesian posterior probability (PP) values on the bottom (only values greater than 0.90 are shown). * indicates that the ML or PP values were not significant at those nodes.
(TIF)

Figure S2 **Phylogram of the *Ophiostoma piceae* and *Ophiostoma minus* species complexes based on βt.** ML phylogram of 28 fungal isolates in the *O. piceae* and *O. minus* species complexes, based on βt. Each strain is indicated following the same criteria as Figure S1. Statistical support for the nodes is shown in the same format as Figure S1.
(TIF)

Figure S3 **Phylogram of the *Sporothrix schenckii-Ophiostoma stenoceras* species complex based on βt.** ML phylogram of 15 fungal isolates in the *S. schenkii*-*O. stenoceras* species complex, based on βt. Each strain is indicated following the same criteria as Figure S1. Statistical support for the nodes is shown in the same format as Figure S1.
(TIF)

Figure S4 **Phylograms of the *Grosmannia aurea* species complex based on βt and EF.** Phylograms of 19 fungal isolates in the *G. aurea* species complex based on βt, and 20 fungal isolates based on EF. Individual strains are indicated by their Genbank accession number or CMW culture collection number (if accession number is not available), and a T if the isolate originates from a species’ type specimen. Isolates associated with *D. valens* that were collected in this study or in the Chinese studies [54,55] are followed by the location they were isolated from in different colors: blue for ENA, green for WNA, and orange for China. Species are indicated between the βt and EF phylograms, with dashed lines connecting the isolates to their species names.
(TIF)

Figure S5 **Phylograms of the *Grosmannia galeiformis* and *Grosmannia olivacea* species complexes based on βt and EF.** Phylograms of 23 fungal isolates in the *G. galeiformis* and *G. olivacea* species complexes based on βt, and 20 fungal isolates based on EF. Each strain is indicated following the same criteria as Figure S4. Statistical support for the nodes is shown in the same format as Figure S4.
(TIF)

Figure S6 **Phylograms of the *Leptographium lundbergii* and *Grosmannia huntii* species complexes based on βt and EF.** Phylograms of 25 fungal isolates in the *L. lundbergii* and *G. huntii* species complexes based on βt, and 23 fungal isolates based on EF. Each strain is indicated following the same criteria as Figure S4. Statistical support for the nodes is shown in the same format as Figure S4.
(TIF)

Figure S7 **Phylograms of the *Leptographium procerum* species complex based on βt and EF.** Phylograms of 27 fungal isolates in the *L. procerum* species complex based on βt, and 28 fungal isolates based on EF. Each strain is indicated following the same criteria as Figure S4. Statistical support for the nodes is shown in the same format as Figure S4.
(TIF)

Table S1 **Collection information for *Dendroctonus valens* collected in ENA and WNA.** ^1^Collections made by Forest Service employees on National Forests are categorically excluded from permit requirements as long as they are limited in extent, are for research purposes, and involve organisms that are not protected by federal, state, or local law [63].
(XLS)

Table S2 **Substitution models and burn-in values for phylogenetic analyses.**
(XLS)

Table S3 **Numbers of isolates of each species collected in each state in ENA and WNA, as well as China.** ^1^The numbers of isolates are followed by the percentage of total isolates that species represents in each location.
(XLS)
