# Supplementary figures and images for: Large Shift in Symbiont Assemblage in the Invasive Red Turpentine Beetle
Source: PLoS One. 2013 Oct 18;8(10):e78126. doi: 10.1371/journal.pone.0078126 (PMC3799831; doi:10.1371/journal.pone.0078126)

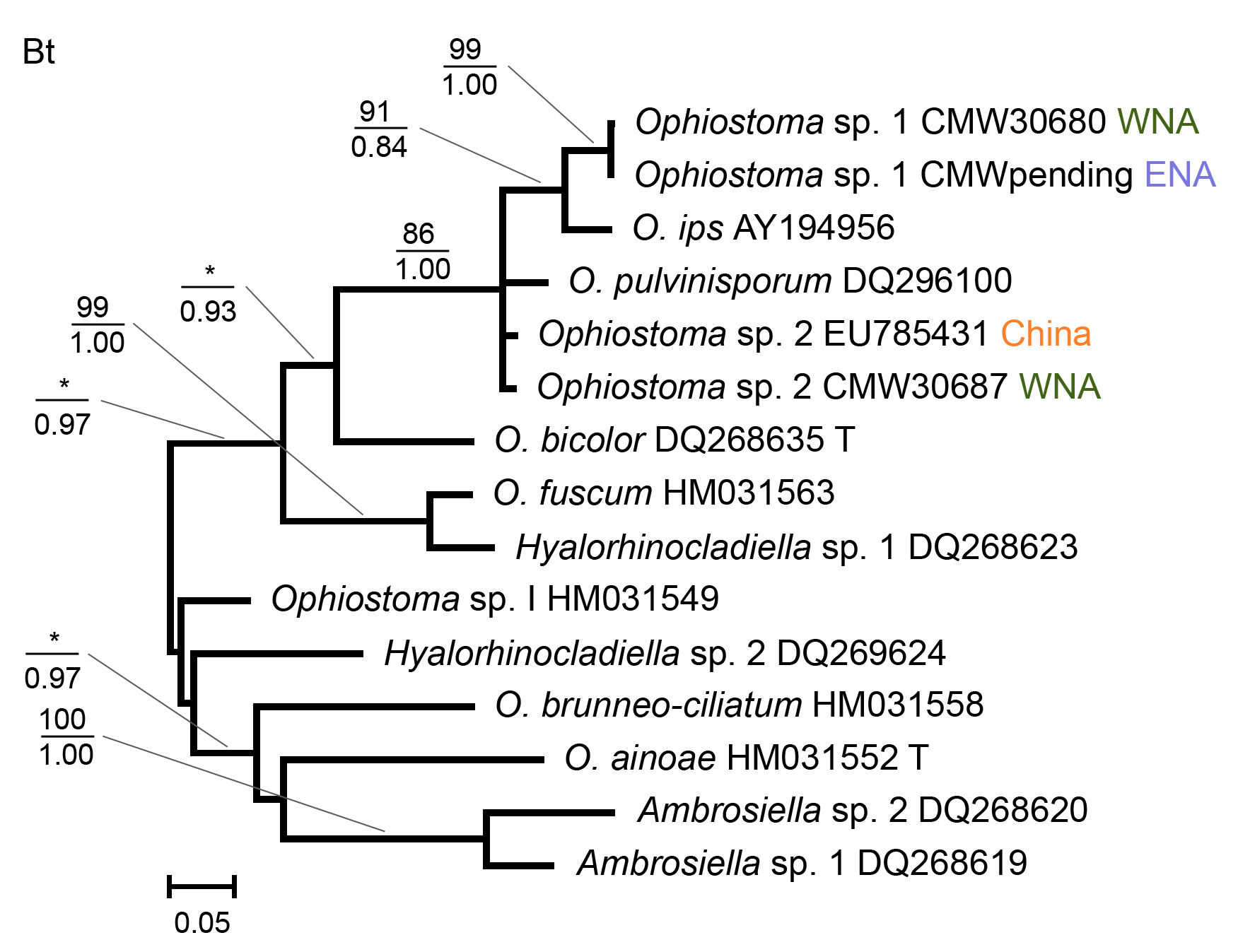

Supplement: Figure S1 — Phylogram of the Ophiostomaips species complex based on βt. ML phylogram of 15 fungal isolates in the O. ips species complex, based on βt. Individual strains are indicated by their species name, followed by their Genbank accession number or CMW culture collection number (if accession number is not available), and a T if the isolate originates from a species’ type specimen. Isolates associated with D. valens that were collected in this study or in the Chinese studies [54,55] are followed by the locations they were isolated from in different colors: blue for ENA, green for WNA, and orange for China. Statistical support is given to the left of the nodes, with ML bootstrap proportions on top (only values greater than 75 are shown), and Bayesian posterior probability (PP) values on the bottom (only values greater than 0.90 are shown). * indicates that the ML or PP values were not significant at those nodes. (TIF) [file pone.0078126.s001.tif]

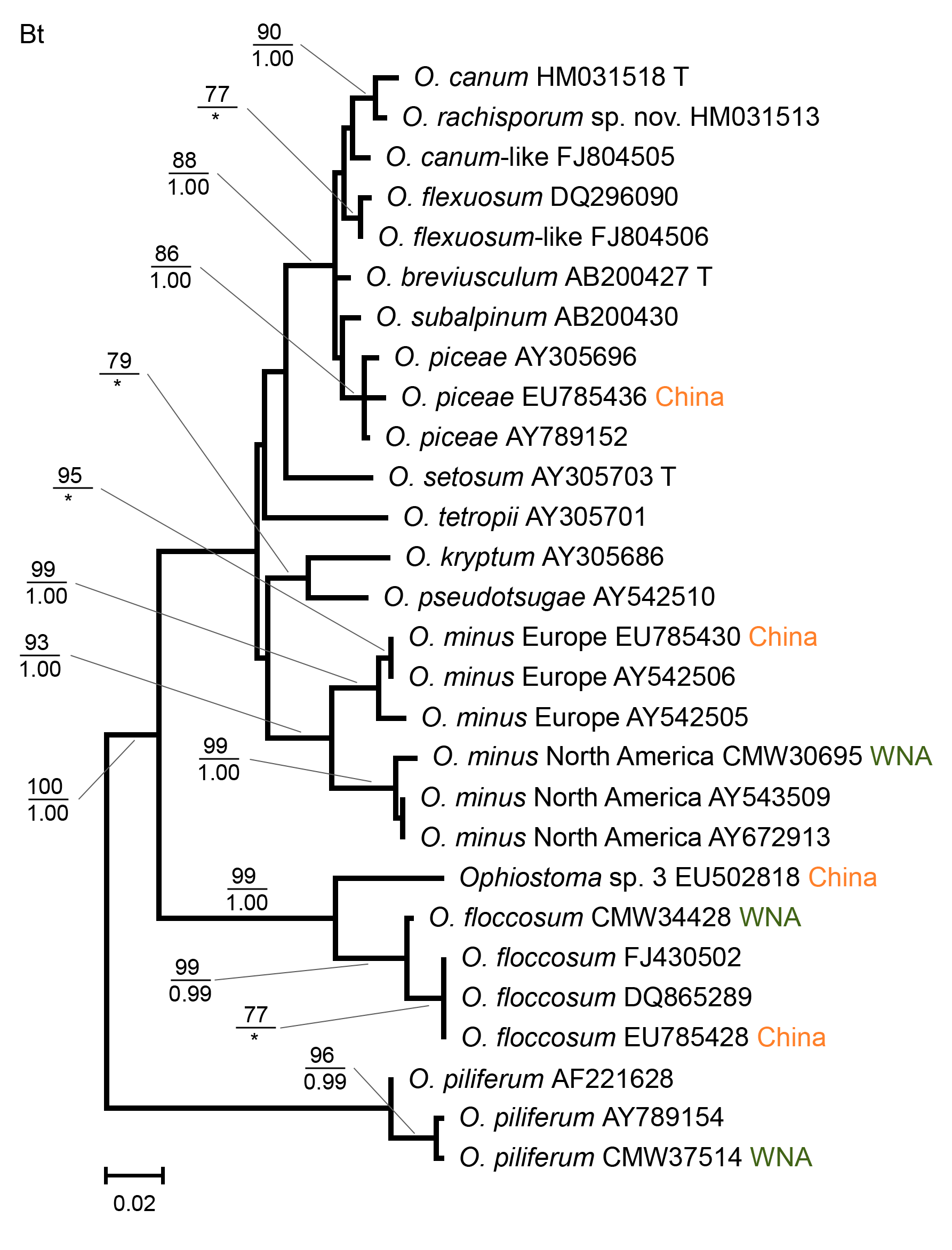

Supplement: Figure S2 — Phylogram of the Ophiostoma piceae and Ophiostoma minus species complexes based on βt. ML phylogram of 28 fungal isolates in the O. piceae and O. minus species complexes, based on βt. Each strain is indicated following the same criteria as Figure S1. Statistical support for the nodes is shown in the same format as Figure S1. (TIF) [file pone.0078126.s002.tif]

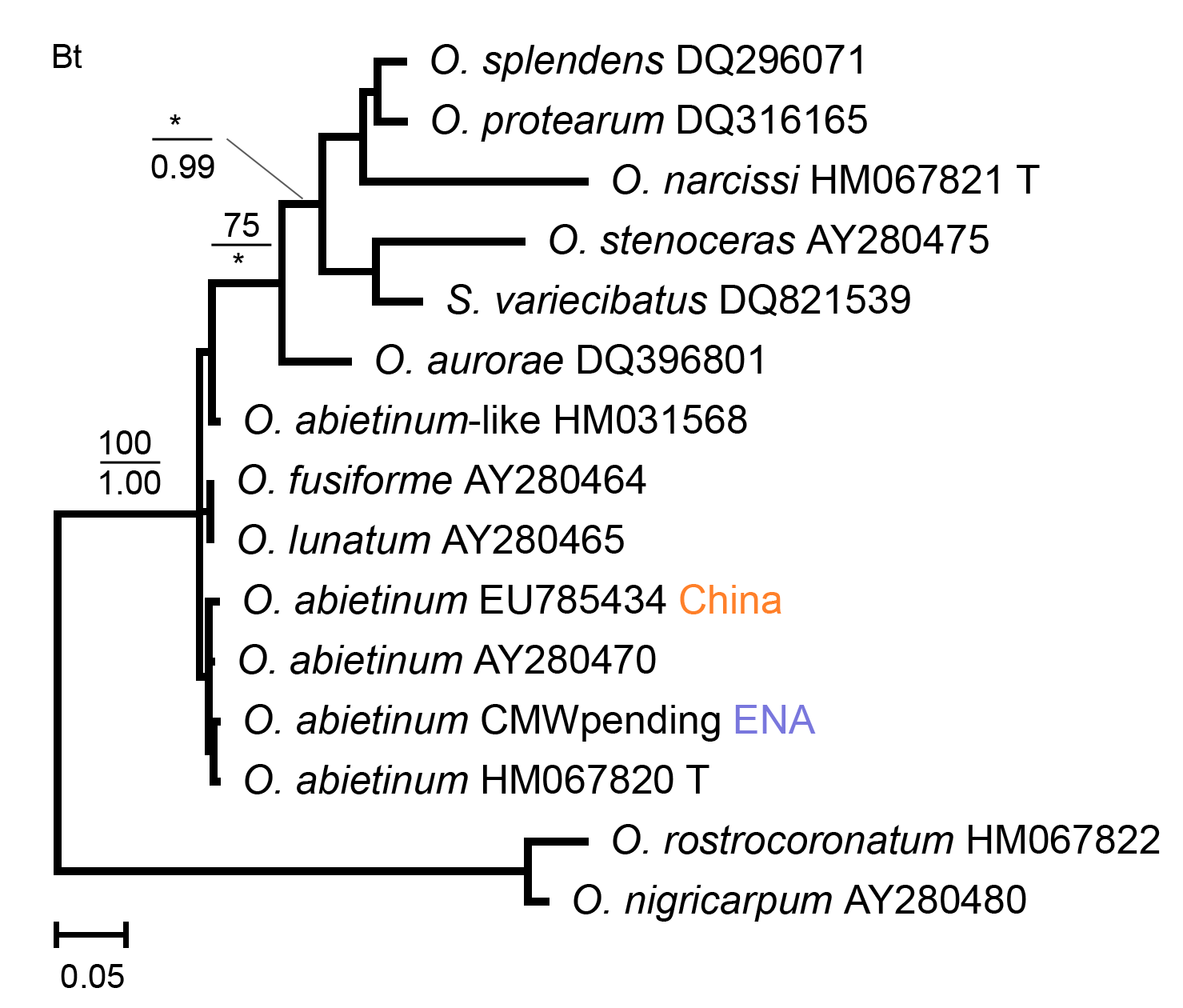

Supplement: Figure S3 — Phylogram of the Sporothrix schenckii-Ophiostoma stenoceras species complex based on βt. ML phylogram of 15 fungal isolates in the S. schenkii-O. stenoceras species complex, based on βt. Each strain is indicated following the same criteria as Figure S1. Statistical support for the nodes is shown in the same format as Figure S1. (TIF) [file pone.0078126.s003.tif]

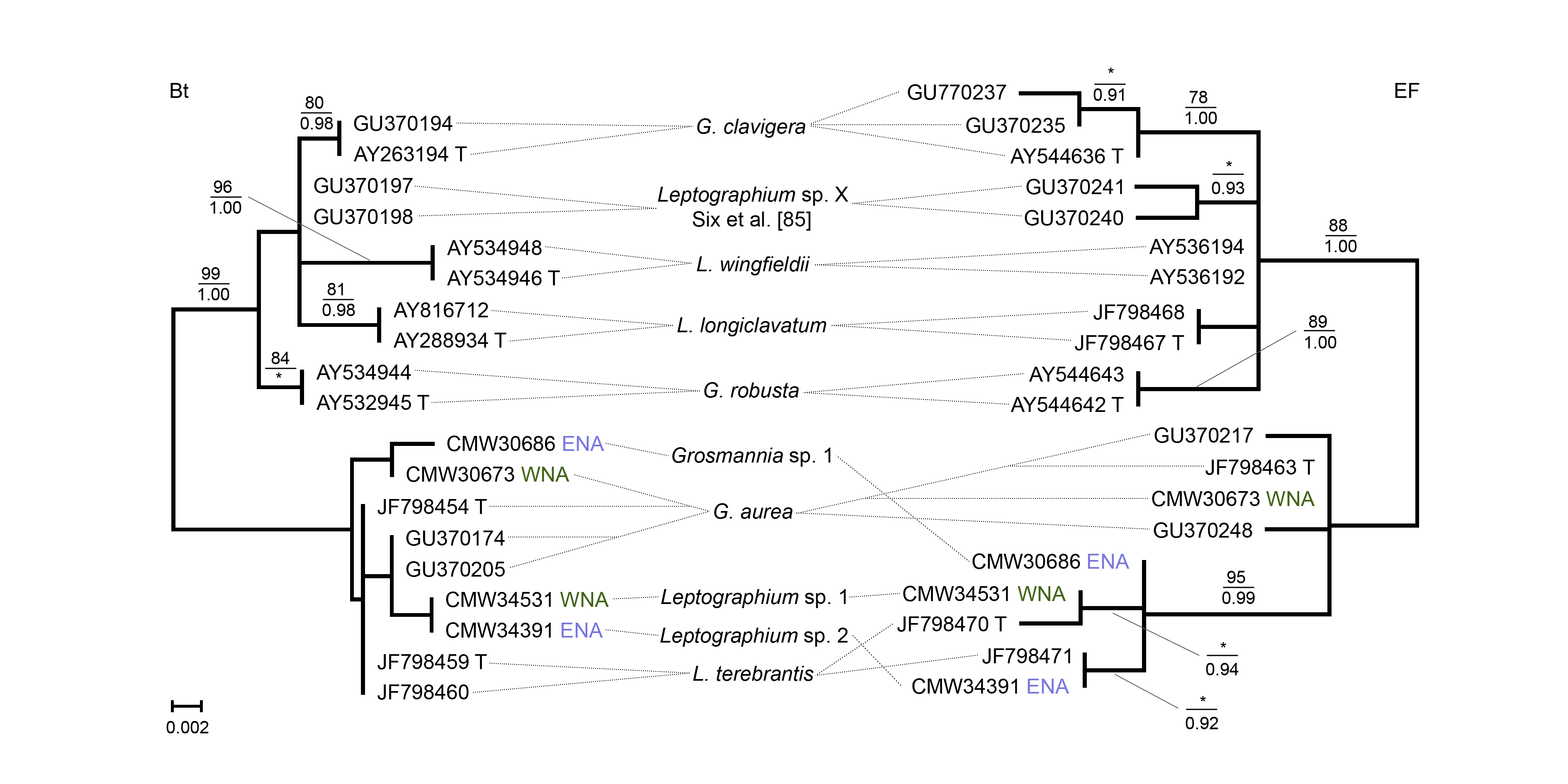

Supplement: Figure S4 — Phylograms of the Grosmannia aurea species complex based on βt and EF. Phylograms of 19 fungal isolates in the G. aurea species complex based on βt, and 20 fungal isolates based on EF. Individual strains are indicated by their Genbank accession number or CMW culture collection number (if accession number is not available), and a T if the isolate originates from a species’ type specimen. Isolates associated with D. valens that were collected in this study or in the Chinese studies [54,55] are followed by the location they were isolated from in different colors: blue for ENA, green for WNA, and orange for China. Species are indicated between the βt and EF phylograms, with dashed lines connecting the isolates to their species names. (TIF) [file pone.0078126.s004.tif]

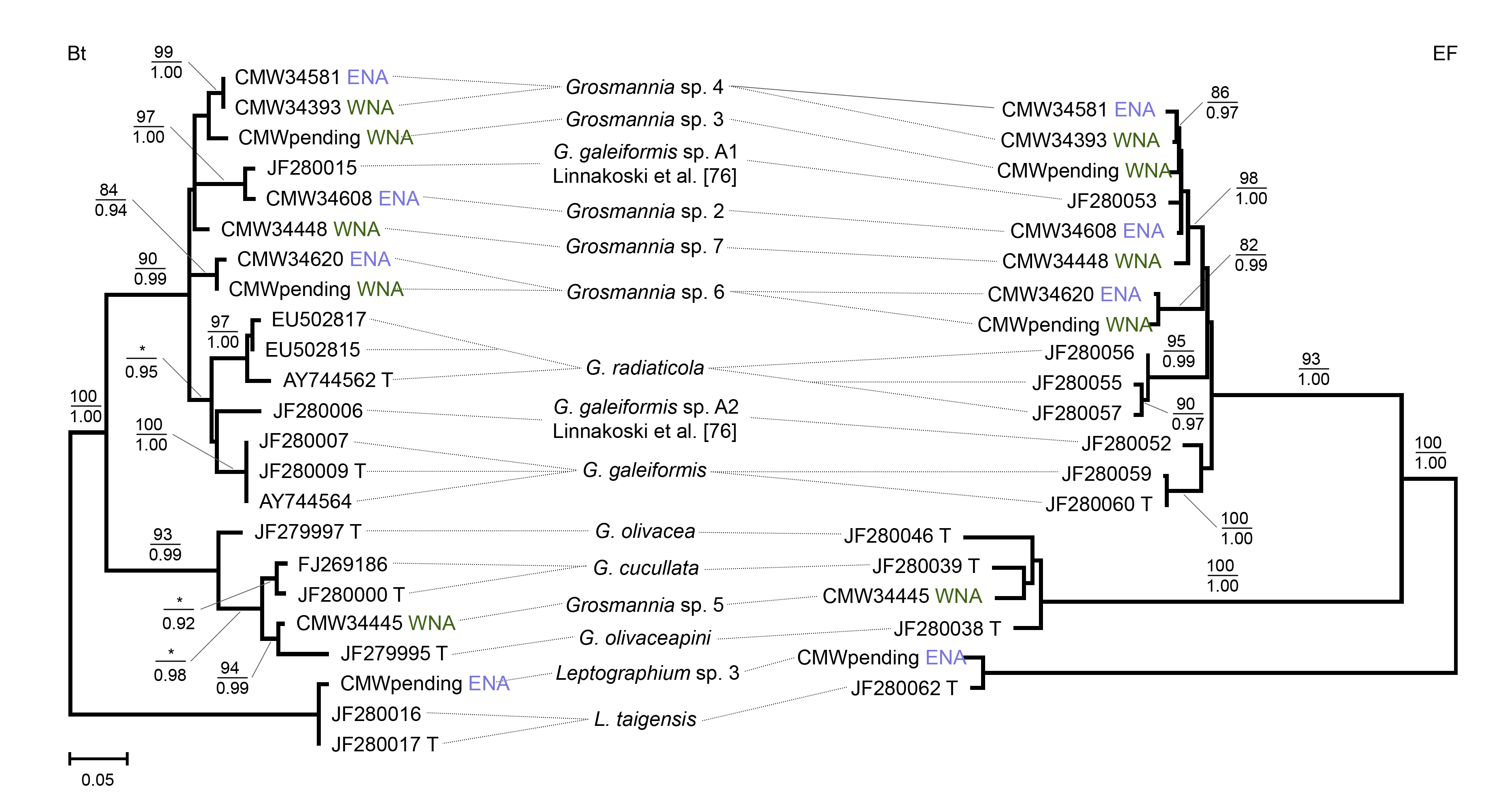

Supplement: Figure S5 — Phylograms of the Grosmannia galeiformis and Grosmannia olivacea species complexes based on βt and EF. Phylograms of 23 fungal isolates in the G. galeiformis and G. olivacea species complexes based on βt, and 20 fungal isolates based on EF. Each strain is indicated following the same criteria as Figure S4. Statistical support for the nodes is shown in the same format as Figure S4. (TIF) [file pone.0078126.s005.tif]

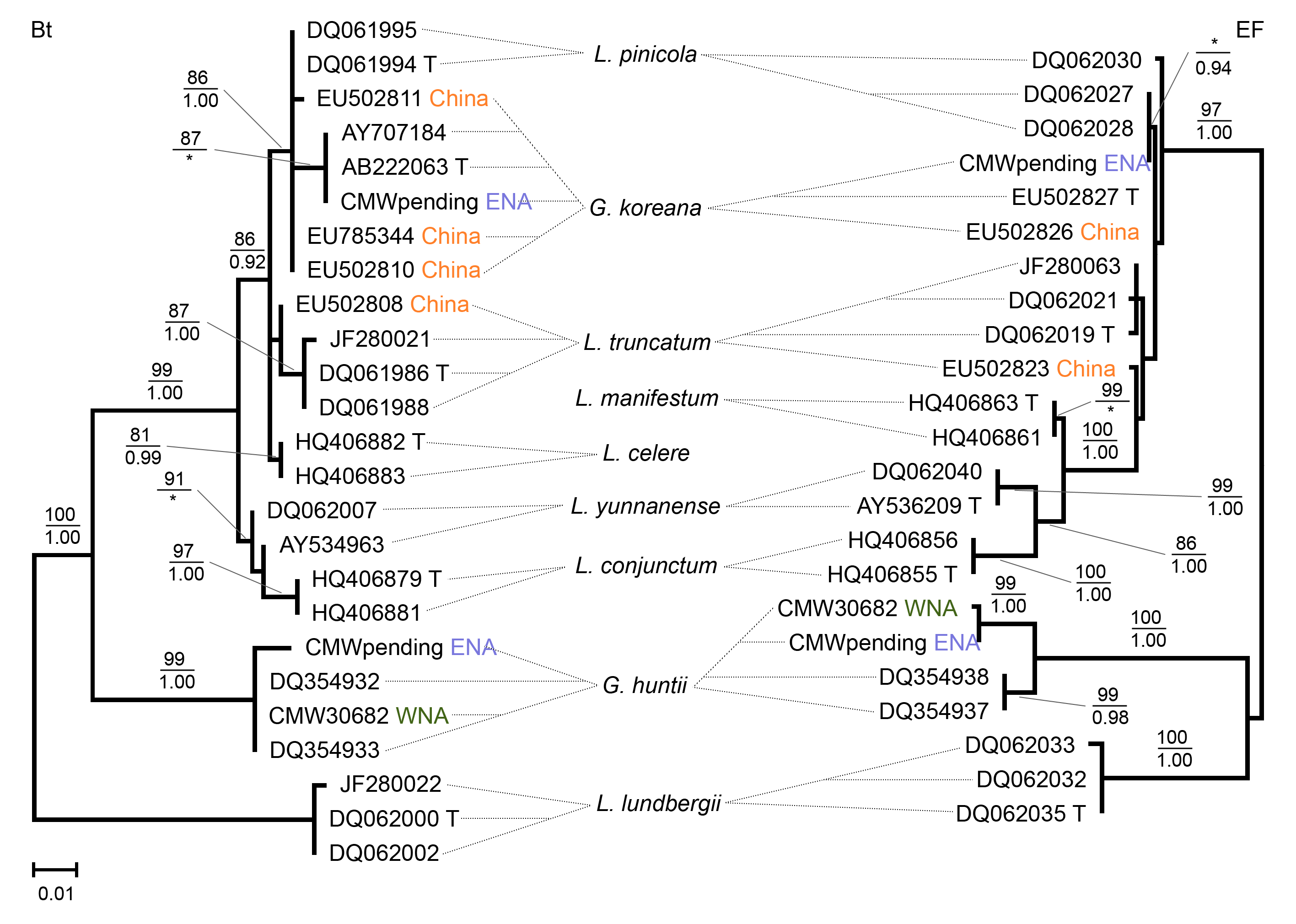

Supplement: Figure S6 — Phylograms of the Leptographium lundbergii and Grosmannia huntii species complexes based on βt and EF. Phylograms of 25 fungal isolates in the L. lundbergii and G. huntii species complexes based on βt, and 23 fungal isolates based on EF. Each strain is indicated following the same criteria as Figure S4. Statistical support for the nodes is shown in the same format as Figure S4. (TIF) [file pone.0078126.s006.tif]

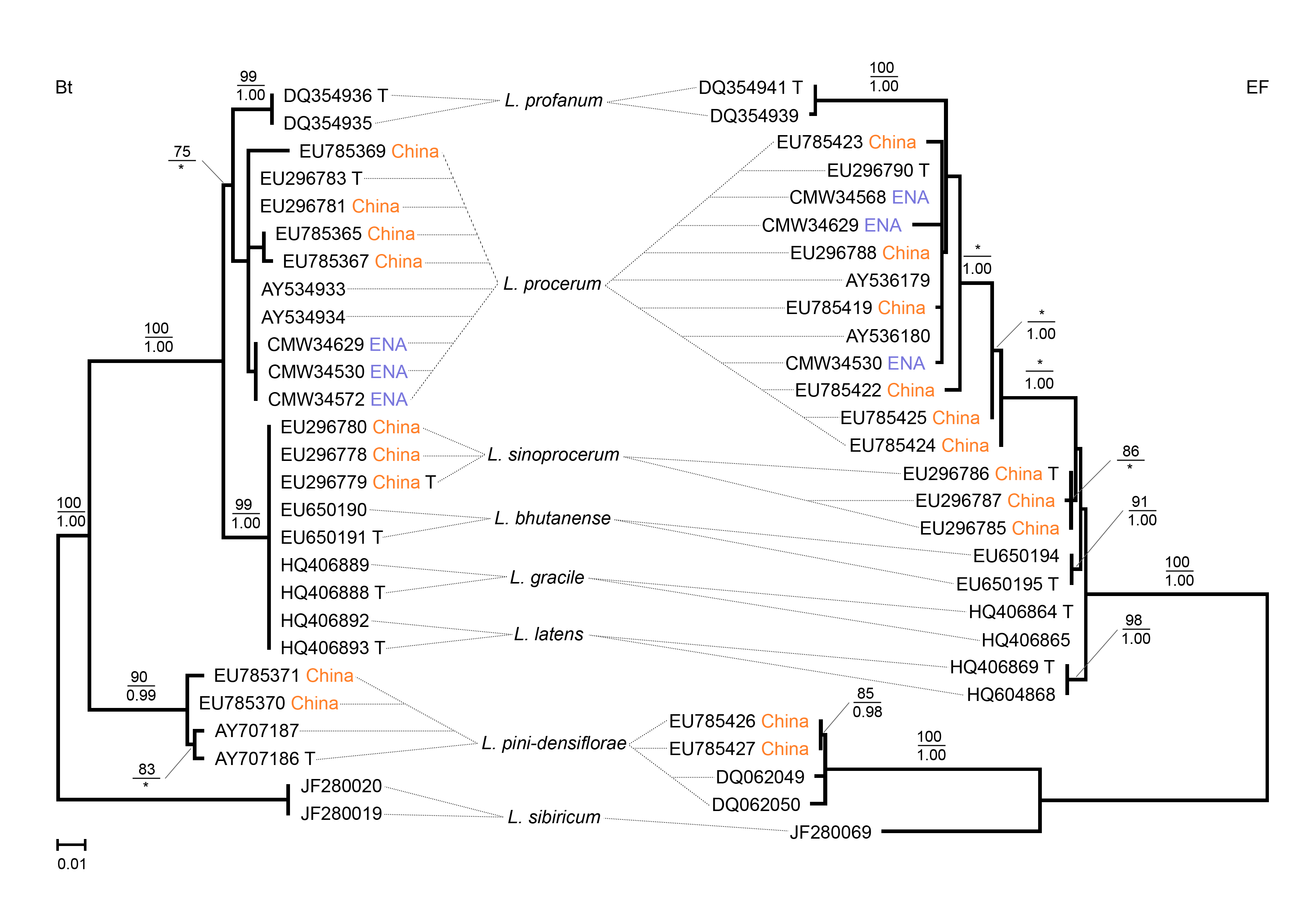

Supplement: Figure S7 — Phylograms of the Leptographium procerum species complex based on βt and EF. Phylograms of 27 fungal isolates in the L. procerum species complex based on βt, and 28 fungal isolates based on EF. Each strain is indicated following the same criteria as Figure S4. Statistical support for the nodes is shown in the same format as Figure S4. (TIF) [file pone.0078126.s007.tif]
